# Five new species and three new females of the genus *Endotricha* Zeller from China (Lepidoptera, Pyralidae, Pyralinae)

**DOI:** 10.3897/zookeys.214.3307

**Published:** 2012-08-07

**Authors:** Yongling Sun, Houhun Li

**Affiliations:** 1 Department of Biology, Dezhou University, Dezhou 253023, Shandong Province, R. P. China; 2 College of Life Sciences, Nankai University, Tianjin 300071, P. R. China

**Keywords:** Lepidoptera, Pyralidae, Pyralinae, *Endotricha*, taxonomy, new species, China

## Abstract

Five new species of the genus *Endotricha* Zeller are described from China: *Endotricha dentiprocessa* **sp. n.**, *Endotricha unicolor* **sp. n.**, *Endotricha shafferi* **sp. n.**, *Endotricha convexa* **sp. n.** and *Endotricha whalleyi* **sp. n.** Females of three species are described for the first time: *Endotricha hoenei* Whalley, 1963, *Endotricha luteogrisalis* Hampson, 1896 and *Endotricha simipunicea* Wang & Li, 2005. Photographs of the adults and both male and female genitalia are provided.

## Introduction

The genus *Endotricha* Zeller, 1847 was erected for the type species *Pyralis flammealis* [Denis & Schiffermüller], 1775. It belongs to the tribe Endotrichini of the subfamily Pyralinae, which includes seven genera and are characterized by having Rs anastomosed with Sc+R_1_ in the hindwing (Solis and Shaffer 1999). Solis and co-authors discussed the structures and the systematic position of the tribe, the subfamily and family (Solis and Mitter 1992, Solis and Shaffer 1999, Solis and Metz 2011).

**Map 1.**
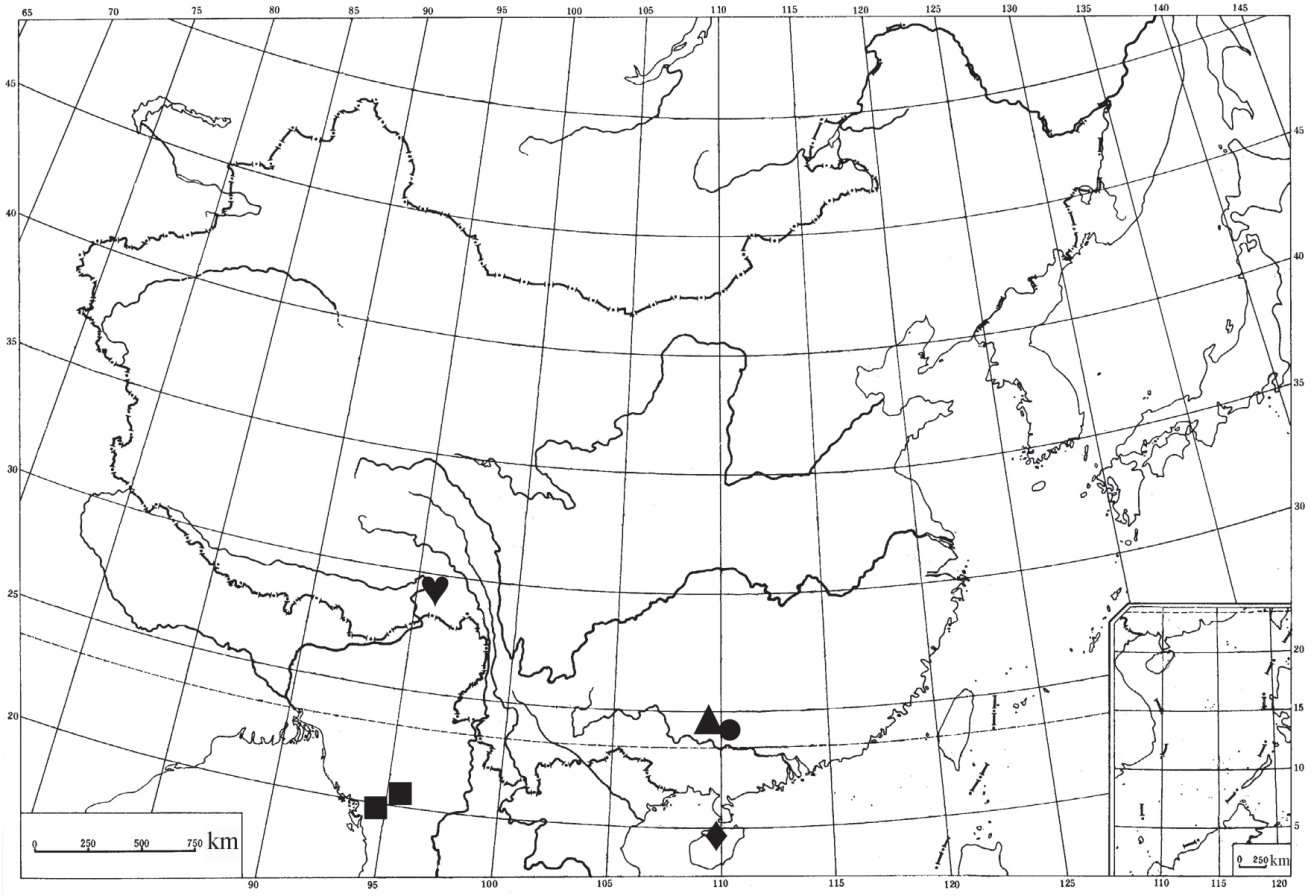
Distribution of new *Endotricha* species in China. ▲ *Endotricha dentiprocessa* sp. n. ■ *Endotricha unicolor* sp. n. ● *Endotricha shafferi* sp. n. ♦ *Endotricha convexa* sp. n. ♥ *Endotricha whalleyi* sp. n.

*Endotricha* is characterized by the forewing usually having a dark-colored ground coloration, the male gnathos being flat and plate-like, and the female corpus bursae having a spined signum. Currently, it comprises over one hundred species worldwide (Whalley 1963, Yoshiyasu 1987 and 1989, Solis and Shaffer 1999, Kirpichnikova 2003, Wang and Li 2005, Lee et al. 2007, Sun and Li 2009, Nuss et al. 2003–2011), occurring throughout the Old World. Thirty-three species were recorded in China prior to this study (Wang and Li 2005, Sun and Li 2009). The aim of the present paper is to describe five new species and report the females of three species for the first time based on the Chinese specimens collected in Fujian, Guangxi, Guizhou, Hainan and Tibet (Map 1).

## Material and methods

This study is based on the examination of the specimens collected in mountainous areas or nature reserves in China by using light traps. Terminology mainly follows Whalley (1963). Genitalia dissection and mounting methods follow Li (2002). Images of the adults were taken with a Nikon D300 digital camera plus macro lens, and the genitalia were prepared with an Olympus C-7070 digital camera. All the examined specimens are deposited in the Insect Collection, College of Life Sciences, Nankai University, Tianjin, China.

## Descriptions of new species

### Endotricha dentiprocessa

Li sp. n.

urn:lsid:zoobank.org:act:07143AC2-7C22-4363-972A-4BC1386712B0

http://species-id.net/wiki/Endotricha_dentiprocessa

Figs 1 9 14

#### Type material.

Holotype ♂ – **China**, **Guangxi Zhuang Autonomous Region:** Qinmu Village, Yongfu County (24°59'N, 109°59'E), 160 m, 5.V.2008, coll. Li Zhang and Hui Zhen, genitalia slide no. SYL11029. Paratypes: 1 ♂, 2 ♀, same data as for holotype except dated 1−5.V.2008.

#### Diagnosis.

This species is superficially similar to *Endotricha costaemaculalis* Christoph, 1881, but can be distinguished in the male genitalia by the valva having the basal 1/4 ventrally dentate and the sacculus being distally produced into a stout thumb-shaped process, and in the female genitalia by the corpus bursae having basal half wider than distal half. In *Endotricha costaemaculalis*, the valva is not dentate ventrobasally, the sacculus process is narrowing to a point; and the basal half of the corpus bursae is narrower than the distal half.

#### Description.

**Figures 1–4.**
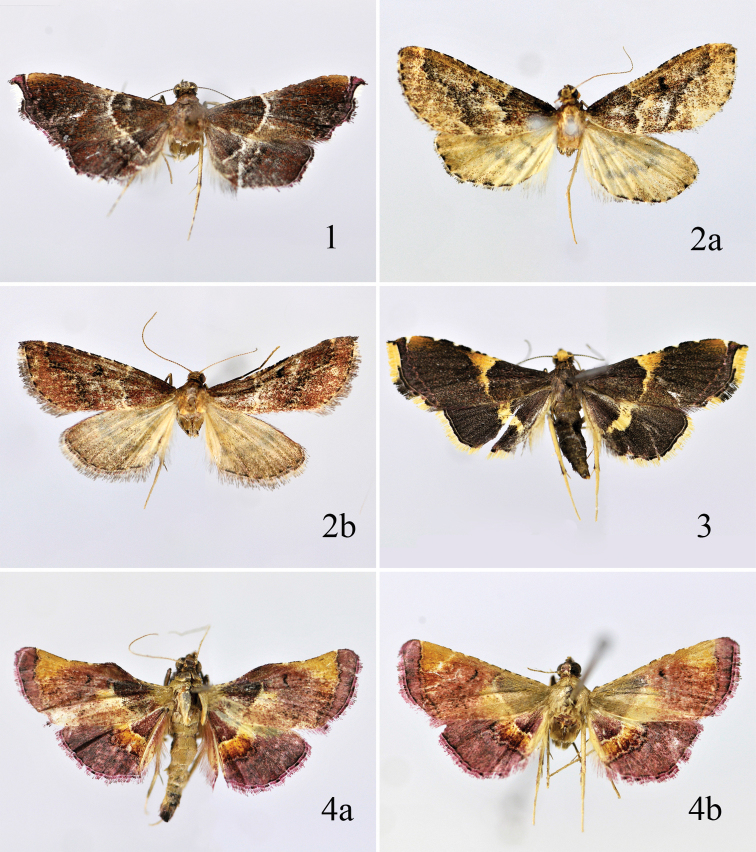
Adults of *Endotricha* spp. **1** *Endotricha dentiprocessa* sp. n., paratype, female **2** *Endotricha unicolor* sp. n. **2a** holotype, male **2b** paratype, female **3** *Endotricha shafferi* sp. n., paratype, female **4** *Endotricha convexa* sp. n. **4a** paratype, male **4b** paratype, female.

Adult (Fig. 1): Wing expanse 17.0−18.5 mm. Head blackish brown. Antenna yellowish brown, scape grayish white dorsoapically, flagellum with blackish brown dorsal annuli. Labial palpus blackish brown except second and third segments grayish-white at apices. Thorax and tegula dark yellowish brown. Forewing blackish brown, covered with dense reddish brown scales; costal margin black, interrupted with white dots, with a large ill-defined yellow spot before subterminal line; antemedian line white, slightly arched outward; discal spot inconspicuous; subterminal line purplish red, discontinuous, edged with black; terminal line black, edged with a broad purplish red band along inner margin; fringe reddish brown mottled black at apex, from below apex to anterior 1/4 of termen creamy white, from anterior 1/4 to tornus black mottled purplish red, blackish grey mottled reddish brown on dorsal margin, with a white basal line. Hindwing concolorous to forewing, yellowish white on costal margin; antemedian line white; postmedian line grayish white, edged with black, sinuate; terminal line black; fringe blackish grey mottled purplish-red and white along termen, grayish white on apex and along dorsal margin. Legs pale yellow on dorsal surface, blackish brown on ventral surface; tarsi with brown rings.

**Male genitalia** (Fig. 9). Uncus inverted triangular, caudal margin gently arched, concave at middle, extending outwards posterolaterally to pointed ends; uncus arm ear-shaped, straight, rounded apically; uncus processes triangular, with dense spines, located at about 2/5. Gnathos narrowly elongate, rounded apically, slightly shorter than arm; arm broad band-shaped. Transtilla large and broad, widened medially, narrowing outwards to both ends. Valva broad tongue-shaped, rounded at apex; costa arched medially; ventral margin concave at base, basal 1/4 with a narrow sclerotized plate bearing large teeth. Sacculus with basal 3/5 broad, with sclerotized narrow edge ventrally, distally produced to a stout thumb-shaped process, slightly hooked backward dorsoapically, reaching beyond middle length of valva apically. Vinculum short and broad; saccus short and broad, triangular. Juxta large hexagonal. Phallus slightly shorter than valva, with fine spines; ductus ejaculation from basal 1/3.

**Female genitalia** (Fig. 14). Ovipositor nearly triangular, narrowing posteriorly to rounded caudal margin. Apophysis posterioris long and slender, about three times length of apophysis anterioris. Ostium bursae broad, trapezoid, weakly sclerotized; antrum heavily sclerotized, funnellike, longer than half length of apophysis anterioris; ductus bursae membranous, shorter than antrum. Corpus burase large and elongate oval, basal half wider than distal half; signum small and rounded, placed at posterior 1/3.

#### Distribution

(Map 1). China (Guangxi).

#### Etymology. 

The specific epithet is from the Latin prefix *dent-*, meaning dentate, and *processus*, meaning process, in reference to the valva with narrow sclerotized plate bearing large teeth ventrobasally.

### Endotricha unicolor

Li sp. n.

urn:lsid:zoobank.org:act:43C9790A-7B57-4108-95B2-EFACBDC8F9D7

http://species-id.net/wiki/Endotricha_unicolor

Figs 2 10 15

#### Type material.

Holotype ♂ – **China, Xizang (Tibet) Autonomous Region:** Milin County (19°10'N, 94°10'E), 2980 m, 30.VII.2010, coll. Houhun Li, genitalia slide no. SYL11147. Paratypes: 2 ♀, same data as for holotype except dated 30−31.VII.2010; 1 ♀, Lulang Town, Linzhi County, 3065 m, 4.VIII.2010, coll. Houhun Li.

#### Diagnosis.

This species is similar in appearance to *Endotricha consobrinalis* Zeller, 1852 from Africa, with some external variation. It can be distinguished in the male genitalia by the apically bluntly rounded uncus arm, the distally dilated gnathos, and by the sacculus produced to a distal-curved process; and in the female genitalia by the oval corpus bursae with signum placed in posterior 1/3. In *Endotricha consobrinalis*, the forewing is ocherous brown, the uncus arm is narrowly rounded, the gnathos is slightly narrowed distally and the sacculus is produced into a distal-straight process; and the corpus bursae is elongate rectangular, and the signum is situated in its posterior 1/4.

#### Description.

Adult (Fig. 2): Wing expanse 20.0 mm. Head yellowish brown. Antenna yellowish brown, with blackish brown dorsal annuli. Labial palpus blackish brown on outer surface, yellowish brown on inner surface, third segment greyish white at apex. Thorax and tegula greyish brown. Forewing brown, irrorate with purple reddish brown scales throughout in female, from base to antemedian line in male; costal margin black, interrupted with white dots, distinct on distal 2/3; antemedian line white, edged with black on inner margin, extending from costal 1/3 obliquely outward to near middle of cell, then inward to fold, and finally arched outward to dorsum; discal spot black, kidney-shaped; postmedian line white, distinctly edged with black on inner margin, extending from about costal 1/8 curved inward to dorsal 3/4; termen with interrupted short black streaks; fringe deep grey. Hindwing yellowish grey, tinged with black distally; ante- and postmedian lines pale silvery grey on dorsal surface, sinuous along both edges, black on ventral surface; termen with black dots and short streaks; fringe greyish brown basally, greyish yellow distally, pale yellow along dorsal margin. Legs yellowish white on dorsal surface, blackish brown on ventral surface.

**Male genitalia** (Fig. 10). Uncus rectangular, gently arched caudally; uncus arm broad, bluntly rounded apically; uncus processes triangular, situated at about 3/5. Gnathos somewhat racket-shaped, rounded at apex. Valva elongately narrow, arched ventrally, rounded at apex; transtilla a curved narrow band. Sacculus elongate triangular, wide basally, tapering to a long spine-shaped process curved distally, apex reaching middle of ventral margin, curved backward. Vinculum broad; saccus short and broad, rounded anteriorly. Juxta broad basally, narrower and nearly parallel distally; lateral lobe short, about 1/3 of its length. Phallus slender and long; ductus ejaculation from basal 1/4.

**Female genitalia** (Fig. 15). Ovipositor nearly triangular, narrowly rounded caudally. Apophysis posterioris long and slender, about 2.5 times length of apophysis anterioris. Ostium bursae broad funnel-shaped, weakly sclerotized; antrum heavily sclerotized, lateral sides nearly parallel, slightly longer than half length of apophysis anterioris; ductus bursae membranous, shorter than antrum. Corpus burase oval; signum small, weak, placed at posterior 1/3.

#### Distribution

(Map 1). China (Tibet).

#### Etymology.

This specific name is from the Latin prefix *uni-*, meaning unitary, and the Latin postfix *-color*, meaning color, in reference to the hindwing without distinct patterns.

### Endotricha shafferi

Li sp. n.

urn:lsid:zoobank.org:act:8757C5F5-B18B-4B8E-A7E0-AA80384B6C16

http://species-id.net/wiki/Endotricha_shafferi

Figs 3 11 16

#### Type material.

Holotype ♂ – **China,** **Guangxi Zhuang Autonomous Region:** Yinsha Protection Station (24°8'N, 110°11'E), Jinxiu County, 700 m, 27.IV.2008, leg. Hui Zhen and Li Zhang, genitalia slide No. SYL11094. Paratypes: 2 ♀, same data as for holotype.

#### Diagnosis.

This species is similarto *Endotricha hoenei* Whalley, 1963. It can be distinguished by the black or deep blackish brown body; the male genitalia with the sacculus squared basally; and the female genitalia with antrum as thick as ductus bursae and having the signum located at posterior 1/3 of corpus bursae. In *Endotricha hoenei*, the body is purplish red; the sacculus is triangular basally; and the antrum is narrower than the ductus bursae.

#### Description.

Adult (Fig. 3): Wing expanse 21.0 mm. Head yellow. Antenna yellowish brown, with blackish brown dorsal annuli. Labial palpus with basal and second segments blackish brown except second segment pale yellow at apex, third segment pale yellow. Thorax and tegula blackish brown. Forewing blackish brown in male, black in female; costal margin with creamy white dots interrupted by short black streaks along distal 2/3, with an ill-defined ocherous yellow patch at inner side of subterminal line; antemedian line yellow, widened to an inverted triangular spot on anterior 1/3, narrowed to beyond dorsal 1/3; discal spot black, small, placed on outer margin of antemedian line; subterminal line purplish reddish brown, sinuate, extending inward to tornus; terminal line black; fringe orange yellow except black at apex and from 1/2 to 3/4 of termen, with a distinct black line at base from anterior 1/4 to before tornus, reddish brown on dorsal margin. Hindwing black; median line yellow, broad, slightly widened medially, narrowed posteriorly; terminal line black; fringe yellow, with a distinct black basal line, pale yellow along dorsal margin. Legs yellow except fore- and midlegs blackish brown on ventral surface.

**Male genitalia** (Fig. 11). Uncus wide at base, narrowed to about middle, then widened to blunt apex; uncus arm triangular, blunt apically; uncus processes ciliiform, placed at about middle. Gnathos short, racket-shaped,rounded at apex. Valva elongately narrow, narrowed basally, rounded apically. Sacculus with basal half squared, distal half suddenly narrowed to a slender rod-shaped process. Vinculum broad, narrowed anteriorly; saccus short, rounded anteriorly. Juxta broad, slightly narrowed letaromedially, with fine satae posteriorly. Phallus slender, about 2/3 length of valva; ductus ejaculation from basal 1/4.

**Female genitalia** (Fig. 16). Ovipositor nearly triangular, narrowly rounded caudally. Apophysis posterioris two times length of apophysis anterioris. Antrum heavily sclerotized, about same size as ductus bursae; ductus bursae membranous, shorter than corpus bursae. Corpus burase elongate oval; signum small, placed at posterior 1/3 of corpus bursae.

#### Distribution

(Map 1). China (Guangxi).

#### Etymology.

This species is named after the late Michael Shaffer (BMNH) in memory of his friendship with the corresponding author as well as for his outstanding work in the taxonomy of Pyraloidea.

### Endotricha convexa

Li sp. n.

urn:lsid:zoobank.org:act:17F0E07F-B69F-4B6D-8848-E6FDD3C71D45

http://species-id.net/wiki/Endotricha_convexa

Figs 4 12 17

#### Type material.

Holotype ♂ – **China**, **Hainan Province:** Yinggeling (19°02'N, 109°50'E), 620 m, 23.V.2010, coll. Bingbing Hu and Jing Zhang, genitalia slide no. SYL11043. Paratypes: 1 ♂, same data as for holotype; 2 ♂, Mt. Wuzhi, 740 m, 14.IV.2009, coll. Qing Jin and Bingbing Hu; 1 ♀, Yinggeling, 30.IX.2010, coll. Bingbing Hu.

#### Diagnosis.

This species is similar to *Endotricha lobibasalis* Hampson, 1906. It can be distinguished by the forewing in male having a hump from costal 1/5 to 2/5; in the male genitalia by the conspicuous gnathos, the ventroapically right-angled valva, and the oval juxta being deeply concave to mid length on posterior margin. In *Endotricha lobibasalis*, the basal 1/3 of the forewing has a gentle hump; the gnathos is inconspicuous, the valva is bluntly angled ventroapically, and the nearly trapezoidal juxta is not concave.

#### Description.

Adult (Fig. 4): Wing expanse 20.0−22.0 mm. Head reddish brown. Antenna yellowish brown, flagellum with blackish brown dorsal annuli. Labial palpus with basal segment reddish brown, second and third segments blackish brown except second segment pale yellow at apex. Thorax and tegula greyish yellow. Forewing purplish red in male, covered with dense black scales on basal 1/3, scattered with black scales on distal 2/3; costal margin with prominent hump extending from 1/5 to about 2/5, then gently concave to before apex, basal 1/3 orange yellow, interrupted with black dots, distal 3/5 black, with yellow spots; large inverted triangular orange yellow patch placed between middle of costal margin and before subterminal line extending downward, its inner margin obliquely extending to middle of subterminal line, outer margin straight, just adjacent to subterminal line; antemedian line yellowish white, slightly arched outward, not reaching anterior margin; discal spot a short strip, black; subterminal line whitish yellow, thin, curved, edged with black on outer margin; terminal line black; fringe purplish red, tinged with black. Hindwing concolorous to forewing except anteriorly whitish yellow from base to distal 1/5; antemedian line whitish yellow, edged with black on inner margin; postmedian line whitish yellow, both margins edged with black, between two lines pale yellow mixed with dense reddish brown scales; terminal line black; fringe purplish red, with blackish brown dots along basal half, pale yellow along dorsal margin. Legs greyish white dorsally, greyish black ventrally, mid tibia with purplish red scales.

Forewing in female greyish yellow from base to antemedian line, from antemedian line to subterminal line yellow with dense reddish brown scales, from subterminal line to apex purplish red; costal margin straight, interrupted with short blackish brown and yellow streaks. Other characters as in male.

**Male genitalia** (Fig. 12). Uncus broad at base, slightly narrowing to 3/4, then broadened to blunt apex; uncus arm broad earlike, extending outwards; uncus processes more or less narrow triangular, set at distal 1/4. Gnathos somewhat racket-shaped, rounded at apex; arm extremely narrow-banded. Valva nearly rectangular, truncate apically, right-angled ventroapically; costa protruding at about distal 1/3, bearing three long reflexed hairs; transtilla broad, uniformly narrow-band. Sacculus wide basally, tapering to a long rod-shaped distal process, apex slightly exceeding middle of valva. Vinculum broad; saccus short and broad, with rounded anterior margin. Juxta more or less oval; posterior margin concave to half length at middle, forming two thumb-shaped lateral lobes. Phallus relatively stout, about same length as sacculus; cornutus conspicuous, about 1/2 length of phallus, toothed; ductus ejaculation from basal 1/3.

**Female genitalia** (Fig. 17). Ovipositor nearly triangular, narrowly rounded caudally. Apophysis posterioris long and slender, about 2.3 times length of apophysis anterioris. Antrum weakly sclerotized, funnel-shaped; bursal ring heavily sclerotized, shorter than half length of antrum; ductus bursae membranous, extremely short. Corpus burase elongate rectangular, length about 3.5 times of width; signum small and rounded, placed medially.

#### Distribution

(Map 1). China (Hainan).

#### Etymology.

The specific epithet is derived from the Latin *convexus*, meaning convex, referring to the forewing hump from costal 1/5 to 2/5.

### Endotricha whalleyi

Li sp. n.

urn:lsid:zoobank.org:act:DB2596C2-912D-41C9-8CBD-79C2E3321647

http://species-id.net/wiki/Endotricha_whalleyi

Figs 5 13

#### Type material.

Holotype ♂ – **China, Xizang (Tibet) Autonomous Region:** Hanmi, Mêdog County (29°13'N, 95°18'E), 2380 m, 9.VIII.2003, coll. Xinpu Wang and Huaijun Xue, genitalia slide no. SYL11155. Paratypes: 1 ♂, same data as for holotype.

#### Diagnosis.

This new species is superficially similar to *Endotricha metacuralis* Hampson, 1916, but can be distinguished by the triangular uncus process placed more posteriorly, the elongate elliptical gnathos not reaching the base of uncus, the elongate triangular sacculus, and the columniform juxta having clustered strong distal spines. In *Endotricha metacuralis* the nearly squared uncus process is placed medially, the larger racket-shaped gnathos reaches the base of uncus, the sacculus is semicircular, and the juxta has weak distal spines.

#### Description.

**Figures 5–8.**
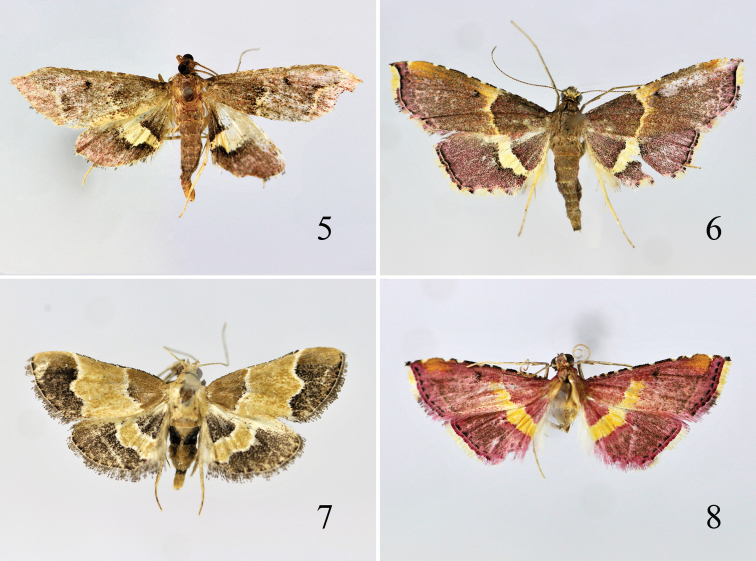
Adults of *Endotricha* spp. **5** *Endotricha whalleyi* sp. n., paratype, male **6** *Endotricha hoenei* Whalley, female**7** *Endotricha luteogrisalis* Hampson, female **8** *Endotricha simipunicea* Wang & Li, male.

Adult (Fig. 5): Wing expanse 16.0 mm. Head greyish brown. Antenna yellowish brown, with blackish brown dorsal annuli. Labial palpus greyish brown except third segment greyish white at apex. Thorax and tegula greyish brown. Forewing greyish brown, irrorated with black and wine reddish scales, almost wine red from postmedian line to termen, costal margin interrupted with yellowish white dots; antemedian line white, edged with black on inner margin posteriorly, extending from beyond costal 1/3 obliquely outward to near middle of cell, then inward to fold, and finally straight to dorsum; discal spot black, conspicuous; subterminal line white, edged with black on inner margin, extending inward from costal margin before apex to the point where M_2_ and M_3_ separated and a right angle formed, then obliquely straight to near tornus; fringe deep grey. Hindwing concolorous to forewing; antemedian line black, not well defined, inconspicuous anterioly; postmedian line thin, black, ill-defined, curved inward and forming a right-angle at distal 1/4 of 1A, then straight to dorsum; broad yellow fascia placed between ante- and postmedian lines, its posterior 1/4 much narrower, handle-shaped; termen interrupted with dark brown dots and short streaks; fringe greyish black basally, yellow distally, pale yellow along dorsal margin. Legs yellowish white on dorsal surface, blackish brown on ventral surface.

**Male genitalia** (Fig. 13). Uncus rectangular, slightly widened before apex, length about 2.5 times width, caudal margin gently arched, naked; uncus arm rounded, stretching outward; uncus processes triangular, with short spines, located at about 3/5. Gnathos elongate elliptical, arm band-shaped. Valva slightly widened medially, distally narrowed to rounded apex; transtilla narrow and straight. Sacculus elongate triangular, produced to a long spine-shaped process, reaching beyond 1/3 of valva. Vinculum narrow; saccus short and broad, rounded anteriorly. Juxta columniform, with clustered stout spines distally. Phallus slender, longer than sacculus; ductus ejaculation from basal 1/4.

**Figures 9–13.**
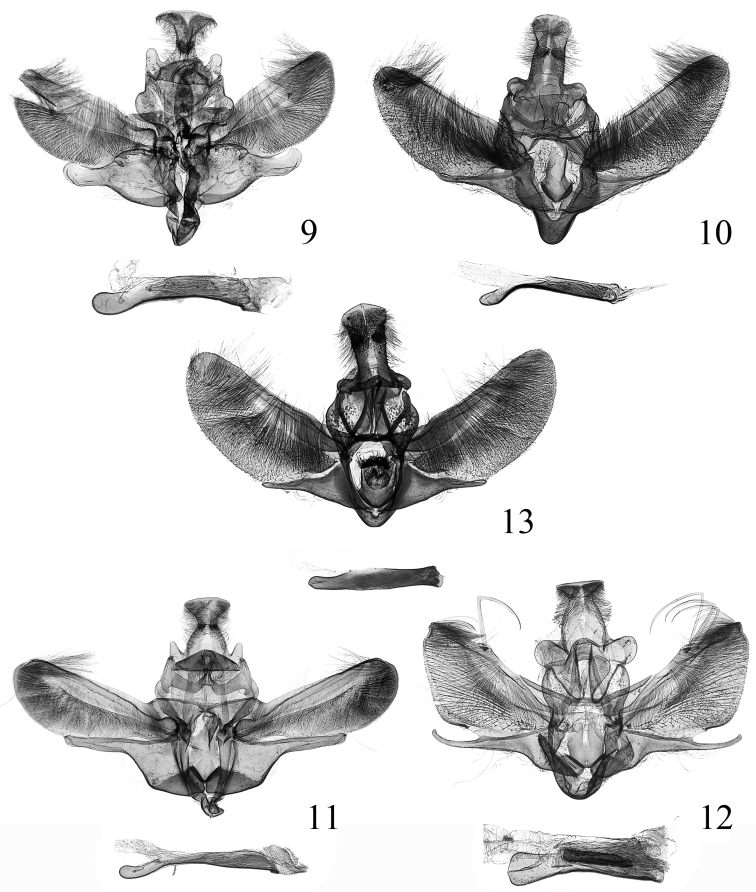
Male genitalia of *Endotricha* spp. **9** *Endotricha dentiprocessa* sp. n., holotype, slide no. SYL11029**10** *Endotricha unicolor* sp. n., holotype, slide no. SYL11147 **11** *Endotricha shafferi* sp. n., holotype, slide no. SYL11094**12** *Endotricha convexa* sp. n., holotype, slide no. SYL11043 **13** *Endotricha whalleyi* sp. n. holotype, slide no. SYL11155.

Female unknown.

#### Distribution

(Map 1). China (Tibet).

**Etymology.** This species is named after the late Paul E. S. Whalley for his contribution to the revision of the world *Endotricha* species.

### Endotricha hoenei

Whalley, 1963

http://species-id.net/wiki/Endotricha_hoenei

Figs 6 18

Endotricha hoenei Whalley, 1963: 430. Holotype ♂, CHINA, Linping, Kwangtung, 18.5.22 (Höne), Brit. Mus. slide No. 6159, deposited in Höne coll, Bonn.

#### Material examined.

2 ♂, 11 ♀, **China, Fujian Province:** Xianfengling, Mt. Wuyi, 1000 m, 26.V.2004, Haili Yu; 1 ♂, 2 ♀, **China**, **Guangxi Zhuang Autonomous Region:** Yinsha Protection Station, Jinxiu County, 700 m, 27.IV.2008, leg. Hui Zhen and Li Zhang; 2 ♂, 6 ♀, Mt. Daming, 125 m, 20.v.2011, coll. Linlin Yang and Yinghui Mou.

#### Description.

Female adult (Fig. 6) with wing expanse 19.0−21.0 mm.

**Female genitalia** (Fig. 18). Ovipositor with basal half nearly parallel laterally, distal half narrowed to rounded apex. Apophysis posterioris about 2.2 times length of apophysis anterioris. Antrum heavily sclerotized, uniformly same thickness, almost as long as apophysis anterioris; ductus bursae membranous, very short. Corpus burase elongate, length about five times of width; signum small and rounded, placed at about anterior 1/3.

**Figures 14–20.**
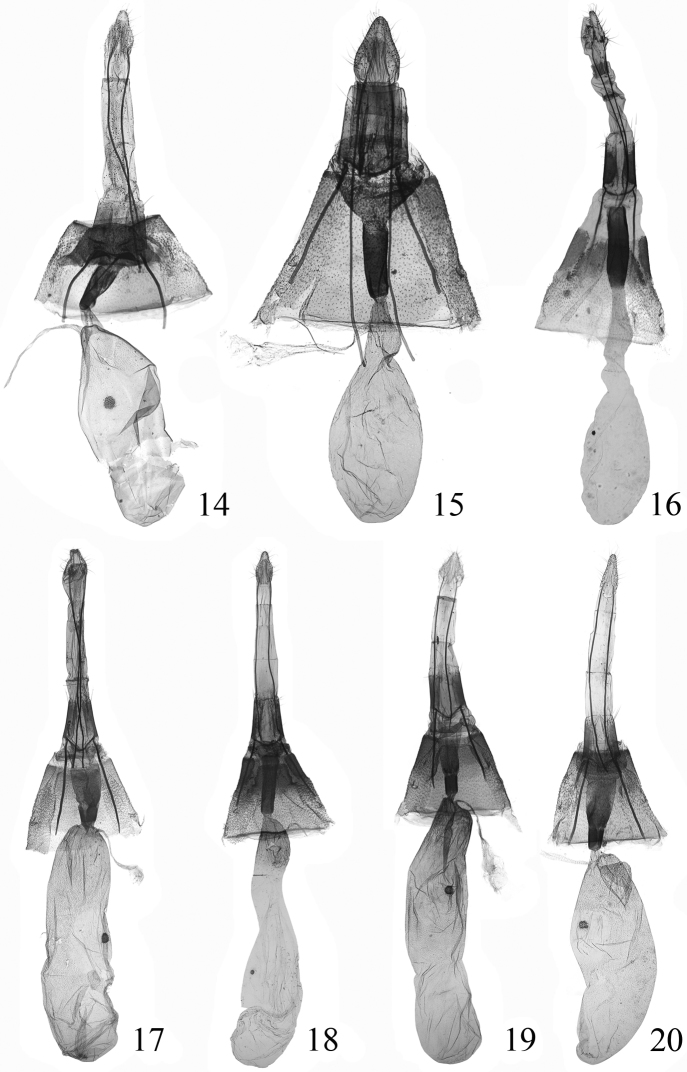
Female genitalia of spp. **14** *Endotricha dentiprocessa* sp. n., paratype, slide no. SYL11030**15** *Endotricha unicolor* sp. n., paratype, slide no. SYL11003**16** *Endotricha shafferi* sp. n., paratype, slide no. SYL11024 **17** *Endotricha convexa* sp. n., paratype, slide no. SYL11112 **18** *Endotricha hoenei* Whalley, 1963, slide no. SYL11109**19** *Endotricha luteogrisalis* Hampson, 1896, slide no. SYL11099 **20** *Endotricha simipunicea* Wang & Li, 2005, slide no. SYL11097.

#### Distribution.

China (Fujian, Guangdong, Guangxi).

#### Remarks.

The female of *Endotricha hoenei* Whalley, 1963 is described here for the first time.

### Endotricha luteogrisalis

Hampson, 1896

http://species-id.net/wiki/Endotricha_luteogrisalis

Figs 7 19

Endotricha luteogrisalis Hampson 1896: 136; Whalley, 1963: 414. Holotype ♂, Bhutan, deposited in Natural History Museum London.

#### Material examined.

4 ♂, 11 ♀, **China, Fujian Province:** Sangang, Mt. Wuyi, 740 m, 25−27.VII.2008, coll. Weichun Li, Yongling Sun and Haiyan Bai; 1 ♀, **China, Hainan Province:** 70 m, 28.V.2007, coll. Zhiwei Zhang and Weichun Li; 1 ♂, Dong’er Work Station, Bawangling, 100 m, 9.IV.2008, coll. Bingbing Hu and Haiyan Bai.

#### Description.

Female adult (Fig. 7) with wing expanse 16.5−20.0 mm.

**Female genitalia** (Fig. 19). Ovipositor nearly triangular, narrowly rounded caudally. Apophysis posterioris more than twice length of apophysis anterioris. Antrum weakly sclerotized, elongate funnel-shaped, about 3/4 length of apophysis anterioris; bursal ring conspicuous, slightly longer than antrum; ductus bursae membranous, very short. Corpus burase elongate rectangular, length four times of width, about 3.5 times as long as apophysis anterioris, posterior 1/5 spinous and granulous; signum rounded, placed at posterior 1/3.

#### Distribution.

China (Fujian, Hainan, Jiangxi, Yunnan).

#### Remarks.

The female of *Endotricha luteogrisalis* Hampson, 1896 is described here for the first time.

### Endotricha simipunicea

Wang & Li, 2005

http://species-id.net/wiki/Endotricha_simipunicea

Figs 8 20

Endotricha simipunicea Wang and Li 2005: 304. Holotype ♂, Mt. Tianmu, Zhejiang Province, alt. 350 m, August 15, 1999, leg. Houhun Li, genitalia slide No. DYL00192, deposited in the Insect Collection, College of Life Sciences, Nankai University.

#### Material examined.

7 ♂, 3 ♀, **China, Fujian Province:** Sangang, Mt. Wuyi, 740 m, 25−27.VII.2008, coll. Weichun Li, Yongling Sun and Haiyan Bai; 1 ♂, **China, Guizhou Province:** Kuankuoshui Nature Reserves, Suiyang County, 1500 m, 14.VIII.2010-VIII-14, coll. Xicui Du.

#### Description.

Female adult (Fig. 8) with wing expanse 13.5−15.0 mm.

**Female genitalia** (Fig. 20). Ovipositor narrow triangular, narrowly rounded caudally. Apophysis posterioris about 2.5 times length of apophysis anterioris. Antrum weakly sclerotized, funnel-shaped; bursal ring conspicuous, shorter than half length of antrum; ductus bursae membranous, very short. Corpus burase elongate oval, slightly narrowed anteriorly; signum small and rounded, toothed, placed at posterior 1/3; ductus seminalis arising from corpus bursae posteriorly.

#### Distribution.

China (Fujian, Guizhou, Zhejiang).

#### Remarks.

The female of *Endotricha luteogrisalis simipunicea* Wang & Li, 2005 is described for the first time.

## Supplementary Material

XML Treatment for Endotricha dentiprocessa

XML Treatment for Endotricha unicolor

XML Treatment for Endotricha shafferi

XML Treatment for Endotricha convexa

XML Treatment for Endotricha whalleyi

XML Treatment for Endotricha hoenei

XML Treatment for Endotricha luteogrisalis

XML Treatment for Endotricha simipunicea
